# The impact of reimbursement systems on equity in access and quality of primary care: A systematic literature review

**DOI:** 10.1186/s12913-016-1805-8

**Published:** 2016-10-04

**Authors:** Wenjing Tao, Janne Agerholm, Bo Burström

**Affiliations:** 1 Centre for Epidemiology and Community Medicine, Stockholm County Council Health Services, Stockholm, Sweden; 2 Department of Public Health Sciences, Karolinska Institutet, Stockholm, Sweden

**Keywords:** Inequality, Healthcare disparities, Socioeconomic factors, Ethnic groups, Health services accessibility, Quality of health care, Outcome assessment, Health policy, Reimbursement mechanisms, Capitation fee

## Abstract

**Background:**

Reimbursement systems provide incentives to health care providers and may drive physician behaviour. This review assesses the impact of reimbursement system on socioeconomic and racial inequalities in access, utilization and quality of primary care.

**Methods:**

A systematic search was performed in Web of Science and PubMed for English language studies published between 1980 and 2013, supplemented by reference tracking. Articles were selected based on inclusion criteria, and data extraction and critical appraisal were performed by two authors independently. Data were synthesized in a narrative manner and categorized according to study outcome and reimbursement system.

**Results:**

Twenty seven articles, mostly from the United States and United Kingdom, were included in the data synthesis. Reimbursement systems seem to have limited effect on socioeconomic and racial inequity in access, utilization and quality of primary care. Capitation might have a more beneficial impact on inequity in access to primary care and number of ambulatory care sensitive admissions than fee-for-service, but did worse in patient satisfaction. Pay-for-performance had little or no impact on socioeconomic and racial inequity in the management of diabetes, cardiovascular diseases, chronic obstructive pulmonary disease, and preventive services.

**Conclusion:**

We found little scientific evidence supporting an association between reimbursement system and socioeconomic or racial inequity in access, utilization and quality of primary care. Overall, few studies addressed this research question, and heterogeneity in context and outcomes complicates comparisons across studies. Further empirical studies are warranted.

**Electronic supplementary material:**

The online version of this article (doi:10.1186/s12913-016-1805-8) contains supplementary material, which is available to authorized users.

## Background

**Fig. 1:**
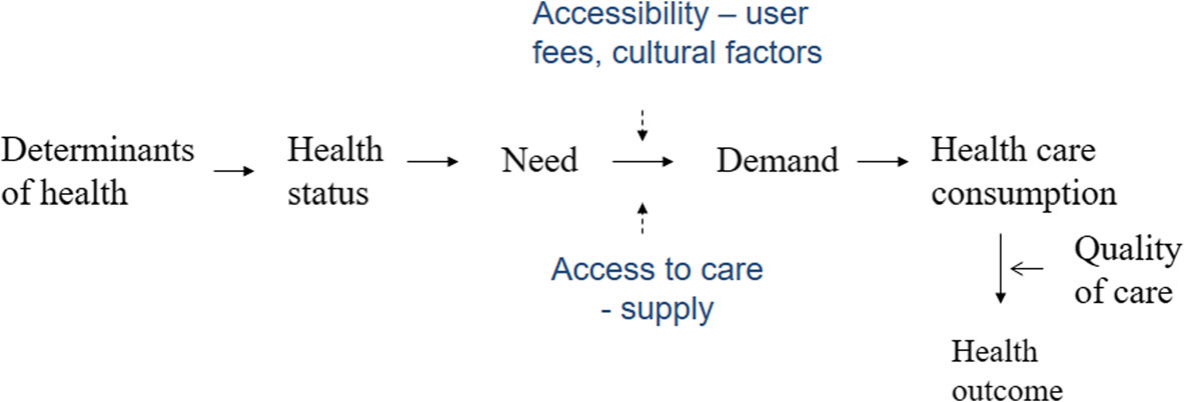
Conceptual framework for socioeconomic and racial inequity in health and health care. Adapted from Burström B. *Int J of Health Services*. 2009, 39(2):271–285 [6]

Socioeconomic and ethnic health inequities are one of the main challenges to public health and well-documented in scientific literature [1]. According to a study of 22 European countries, low socioeconomic status implied higher rates of death and poorer self-assessed health [2]. There are significant differences in chronic disease prevalence and mortality between racial groups, such as cardiovascular disease, as evidenced by studies from North America and the United Kingdom (UK) [3–5]. Although health care can act as a vehicle for reducing health inequalities in the population, there is an abundance of literature indicating the existence of socioeconomic and racial inequity in both access to care and in the quality of care received. Based on the conceptual framework of the health care continuum by Burstrom, inequities may arise at any stage of the continuum [6]. In Fig. 1, we have extended this framework with 'Quality of care' and 'Health outcome' where inequities are also observed. In the United States (US), patients with Medicaid insurance face difficulties in finding healthcare providers willing to accept them [7], and low-income individuals with chronic conditions are less likely to receive standard of care [8]. Fewer Hispanics and African Americans have a regular primary care provider and they are more likely to visit the emergency room. There is a widening racial gap in admissions for ambulatory care sensitive conditions (ACSC), indicating insufficient care in the outpatient setting [9, 10]. In Sweden, high income individuals aged 65+ have significantly more doctor’s visits than low income individuals after adjustment for health status, and non-attendance in breast cancer screening is associated with disadvantaged socioeconomic position [11, 12].

Primary care has a central role in health care delivery, and a strong primary care system has been shown to reduce health inequalities [13, 14]. Funding mechanisms of  primary care might have direct and indirect implications for  service delivery, and reimbursement systems may create incentives to achieve policy objectives, such as improving access to care, quality of care, cost containment and recruitment of physicians to underserved areas [15]. As a result, the setup of reimbursement might ameliorate or aggravate existing health care inequities. Reimbursement through fee-for-service (provider is reimbursed per item of service) might improve access to services, but can also increase the risk of overtreatment [16]. Reimbursement through capitation (provider receives a periodical lump sum per listed patient irrespective of services provided) could encourage cost-effective treatments and preventive services, but providers might be inclined to undertreat patients or choose to list healthier patients that jeopardizes access to care for vulnerable populations [17]. To mitigate some of these effects, reimbursement through capitation may be adjusted for age, socioeconomic factors or disease diagnosis [18]. Reimbursement through pay-for-performance (provider reimbursement is based on process and outcome indicators of clinical relevance) aims to increase quality of care and has been adopted as a complement to other reimbursement practices in several countries [19]. However, concerns have been raised that pay-for-performance might increase inequity if health care providers choose to treat patients that are more likely to achieve favourable outcomes [20].

Available literature addressing the impact of reimbursement systems on health care inequity is inconsistent. A systematic review on the impact of the Quality and Outcomes Framework (QOF), a large pay-for-performance scheme for primary care physicians in the UK, concluded that existing inequalities in chronic disease management largely persisted after implementing pay-for-performance [19]. The impact of fee-for-service and capitation on health care inequalities has not been previously reviewed. Despite this knowledge gap, reimbursement to health care providers has been modified to serve policy purposes. In 2010, a nationwide choice reform in primary care was introduced by county councils and regions in Sweden. The reform enabled private providers to freely establish clinics anywhere and list patients. Concurrently with the reform, each county council implemented different reimbursement systems in primary care. With the increasing use of reimbursement systems as a mean of achieving policy goals, there is a need for empirical evidence to inform policy. The aim of this review is to compare the different types of reimbursement system in relation to socioeconomic and racial inequalities in access, utilization and quality of care.

## Methods

Inequity refers to systematic differences that is created by unjust social processes and avoidable, and is frequently distinguished from inequality [21]. However, in this review we have chosen to use the terms interchangeably due to varying terminologies in the included studies [22].

### Search strategy

We conducted a systematic search of English-language literature published between 1^st^ of January 1980 and 30^th^ of September 2013 in two electronic databases: Web of Science Core Collection and PubMed. The search string was based on the PICO outline referenced by Petticrew et al. and based on index terms and free text terms [23]. Primary health care, reimbursement system, equity and their related synonyms built up the three main components of the search string. The search strategy was adapted between databases using the appropriate controlled vocabulary when applicable. For details of the full search strategy see Additional file 1.

### Study selection

Studies with experimental or observational designs conducted in primary care settings were included in this review. Due to heterogeneity in health care context between low-, middle- and high-income countries, only studies from high-income countries based on the definition of the World Bank Group were selected for this review. The three reimbursement systems assessed were capitation, fee-for-service and pay-for-performance. All patient-related outcomes were of interest. Only original articles evaluating inequalities in relation to socioeconomic position or race/ethnicity were considered for this review; studies that addressed other dimensions of inequality such as age, sex and geographical distribution were excluded. Other exclusion criteria included publications that 1) lacked abstract in the databases; 2) lacked a comparison group to the reimbursement system(s) under study; 3) assessed a non-patient related outcome, e.g. physician satisfaction and total healthcare costs; and 4) when the type of reimbursement system could not be determined from the information given in the article.

Duplicates from the two databases and studies that clearly did not meet the inclusion criteria based on abstract and title were removed through an initial screening. Full-text articles were retrieved for the remaining search results and were independently assessed for eligibility by two authors (WT and JA); any disagreements were resolved after discussion. If a longitudinal study analysed health care inequalities between socioeconomic or ethnic groups separately for each year but did not test if the inequalities differed between years (i.e. difference-in-difference analysis), we concluded that the reimbursement system had an impact on inequity when within-year comparisons were statistically significant for one year but not another. The reference lists of the final selection of studies were screened for articles of potential relevance.

### Data extraction and critical appraisal

The quality of eligible studies was independently assessed by two authors (WT and JA), using instruments developed by Zaza et al. [24] and the University of Manchester [25]. The checklists were selected after reviewing several tools for critical appraisal [26]. Strengths and weaknesses of each study were discussed between the reviewers who assessed the papers and whenever disagreement arose, a third reviewer (BB) was consulted. Overall quality of the papers were divided into high, medium and low based on assessments using the checklists. High risk of bias, confounding and insufficient description of data analysis were weighted more heavily than other items on the checklists. Studies that were deemed to be of low quality were excluded from the final synthesis. Data from the final selection of studies were extracted according to a predetermined data extraction form (see Additional file 2). Findings from the studies were synthesized in a narrative manner using an aggregative approach, [27] and organized according to study outcome (access/utilization of healthcare, quality of care/patient outcome) and reimbursement system (capitation versus fee-for-service, pay-for-performance). Within each category, results from studies addressing the same medical conditions were combined. Due to heterogeneity in context, design and outcome, statistical pooling of study results was not possible.

## Results

**Fig. 2:**
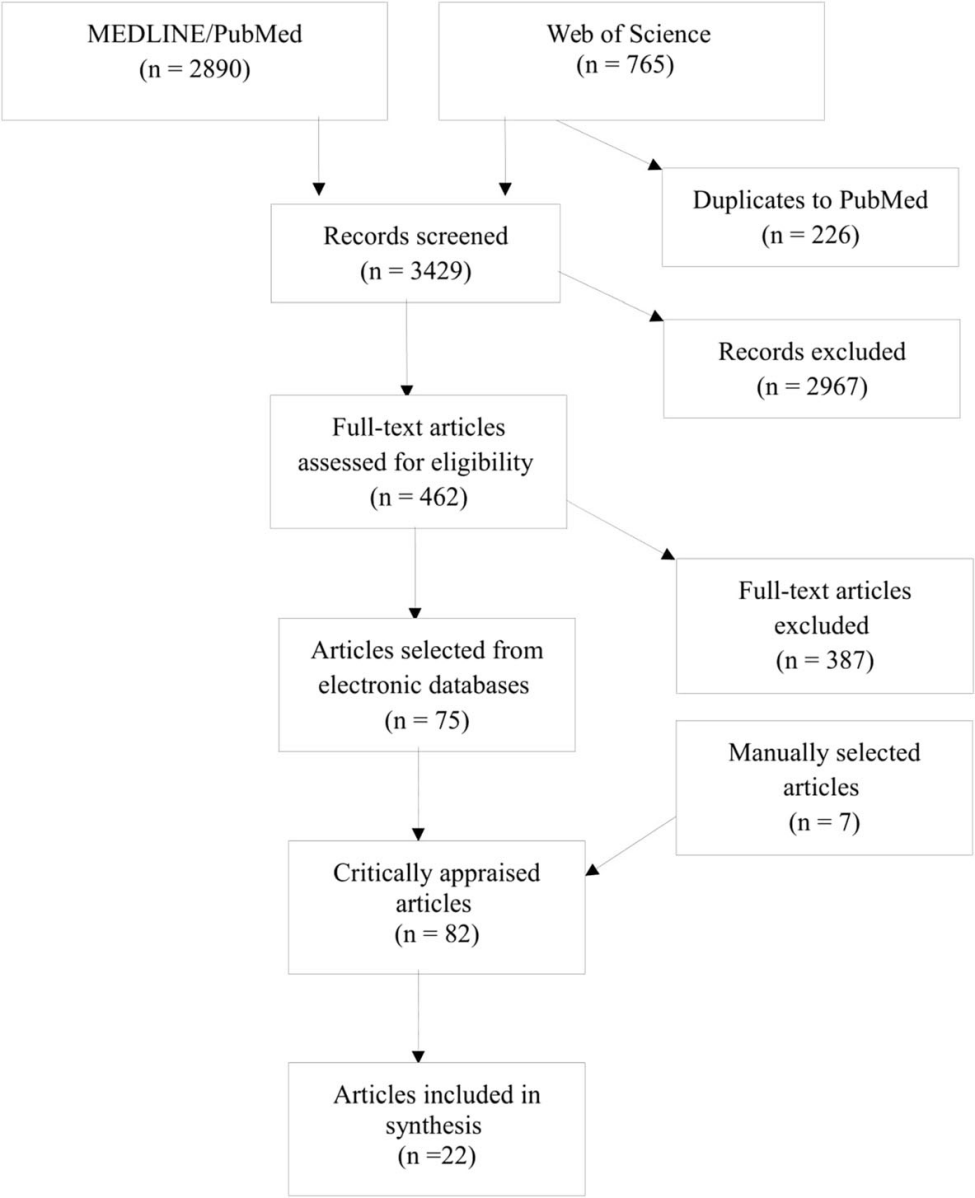
Flow chart for selection of included studies

The search yielded 3,655 articles; 765 in Web of Science and 2,890 in PubMed. Based on title and abstract, we identified 462 articles for full-text assessment and selected 82 for full-text review and critical appraisal. Finally, 22 articles were included in the review based on inclusion/exclusion criteria and quality assessment (Fig. 2). After full-text review and critical appraisal, the most common reasons for exclusion were 1) socioeconomic and racial/ethnic inequities were not addressed (*n* = 19); 2) the study lacked a comparison group for the reimbursement system (*n* = 12); 3) reimbursement system could not be identified (*n* = 10); and 4) the research question did not match the aim of the systematic review (*n* = 10). When two or more studies used the same study population and addressed similar outcomes, we included the most recent publication in this review, which resulted in the exclusion of one study [28]. Two more studies were excluded due to insufficient description of data analysis, [7] and high risk of confounding (study only controlled for sex) [29]. Summaries of each included article are described in Additional file 3. We chose to keep the terminology consistent with the referenced studies. Thus, the terms race and ethnicity are presented interchangeably according to what was used by the author in the articles.

### Capitation versus Fee-for-service

Seven studies compared capitation to fee-for-service. Most studies were from the US and addressed outcomes related to health care access and utilization. Overall findings from the studies are summarized in Table 1.

**Table 1:** Summary of study results comparing capitation to fee-for-service in regards to socioeconomic or racial inequity in admissions for ambulatory care sensitive conditions, access to primary care and patient satisfaction

	Fee-for-service	Capitation
Ambulatory care sensitive admissions	Reference	0/+
Access to primary care	Reference	+
Patient satisfaction	Reference (non-capitation)	0/-

“0” indicates no difference in inequity, “-“ indicates greater inequity, and “+” indicates lesser inequity. “0/+” and “0/-“ indicate that results were mixed depending on outcome and/or socioeconomic or ethnic/racial group. Values from fee-for-service was used as the reference to which values from capitation was compared

### Patient satisfaction

One study assessed the impact of reimbursement system on patient satisfaction. In a nationwide telephone survey in the US, participants were asked to rank their last visit to the primary care physician [30]. Minority groups (Black, Hispanic, and Native American/Asian/Pacific Islander) reported lower satisfaction than whites in a capitated insurance plan than in a non-capitated insurance plans, but the difference was only significant for physician’s ability to listen and to explain among English-speaking Hispanics.

### Access to healthcare

A US study found that there were less racial differences in access and utilization of care among Medicaid beneficiaries under managed care insurance plans (primarily capitation) than under fee-for-service plans. Differences in the proportion of enrolees who had a usual source of care, had visited any doctor in the last year, or had used ER in the last year, were significantly smaller between blacks and whites on managed care than fee-for-service plans. Similar results were observed for Hispanics in regards to having a regular source of care [10]. The beneficial impact of managed care on health care inequalities were confirmed in another study of Medicare patients using two different surveys. Compared to fee-for-service, managed care increased the likelihood of Hispanics having a usual source of care relative to white enrolees, and blacks were more likely to have seen a health professional recently. However, there was no significant difference between managed care and fee-for-service in regards to racial disparities in delaying care for cost reasons, obtaining necessary medical care, or seeing a medical specialist or general practitioner during the year [31]. A survey study of 137 primary care practices in Canada found that immigrants generally consumed more primary care than Canadian-born participants. However, among immigrants who had resided in the country less than 5 years, those who attended fee-for-service practices consumed a lesser amount of health care and experienced inferior access to primary care than Canadian-born [32].

### Ambulatory care sensitive conditions

Higher rates of ACSC admissions indicate limited access to and lower quality of primary care [33]. Bindman et al. compared ACSC admissions among Medicaid fee-for-service enrolees to Medicaid managed care enrolees in California between 1995 and 1999 [9]. The study found that ACSC admission rates were lower in managed care patients than fee-for-service patients, and the difference in admission rates between reimbursement plans was greater for African American, Asian and Latino groups than whites. Distinctions were made between voluntary and mandatory managed care enrolees, and the authors noted that the lower rates of ACSC admissions persisted in both groups and across minorities [9].

### Pay-for-performance

A majority of the studies on pay-for-performance evaluated the impact of QOF on quality of care. According to this scheme, general practitioners in the UK are rewarded for achieving predetermined targets that represents up to 25 % of the practices’ income [34]. The studies included in this review addressed disease-specific and composite outcomes, and the overall findings from pre- and post-implementation are summarized in Table 2.

**Table 2:** Summary of study results comparing inequity in the management of clinical conditions  before and after the implementation of pay-for-performance

Pay-for-performance	Before	After
Diabetes	Reference	0/-
Cardiovascular disease	Reference	0/-
Respiratory disease	Reference	0
Multiple diagnosis	Reference	0
Preventive care	Reference	0

“0” indicates no change in inequity, “-“ indicates increased inequity and “0/-“ indicate that results were mixed depending on outcome and/or socioeconomic or ethnic/racial group. Values obtained after the implementation of pay-for-performance were compared to the baseline values (reference)

### Diabetes

Alshamsan et al. compared the change in diabetes-related measurements for three time periods, i.e. pre-QOF, immediately after the introduction of QOF, and post-QOF [35]. The study noted that levels of mean HbA1c, total cholesterol and mean systolic blood pressure were decreasing in all ethnic groups (white, black and South Asian) prior to QOF, and the new scheme did not seem to add any additional benefit to this downwards going trend. The existing ethnic disparities between whites, blacks and South Asians remained largely unchanged after the introduction of QOF.

In a study by Millett et al., black Caribbean, black African, Indian, Pakistani and Bangladeshi diabetic patients achieved target HbA1c to a lower extent than whites. They were also more often prescribed oral hypoglycemic agents than whites, but were less likely to receive insulin. The combination of these observations indicates that suboptimal treatment of disease was more common in these two minority groups [36]. No differences were, however, observed for other ethnic groups. The rate of prescription for lipid-lowering drugs and antihypertensive drugs improved among diabetes patients across ethnicities post-QOF, but it had little impact on reducing pre-existing health inequalities. It is unclear if the improvements in prescription rate could be attributed to QOF alone, as the study did not estimate the underlying trend of change in prescription rates over time [36].

In a Scottish study, diabetic patients from the least deprived quintile were significantly more likely to achieve target HbA1c levels compared to the most deprived quintile, and this difference remained unchanged after the introduction of QOF [37]. In contrast, the proportion of diabetic patients that reached target values of cholesterol was higher in the most deprived quintile than in the least deprived quintile whereas no difference was observed for target systolic blood pressure. Overall, pay for performance seemed to have little impact on socioeconomic inequalities in intermediate clinical outcomes among diabetes patients [37].

According to a large cohort study from the UK, QOF improved care for all diabetes patients (the proportion of patients achieving target values of blood pressure, total cholesterol and HbA1c exceeded the expected levels based on extrapolation of prior trends), but the pay-for-performance scheme seemed to have little impact on reducing socioeconomic differences [38]. Differences in blood pressure levels between the least and most deprived quintiles were attenuated after the introduction of QOF, but the opposite trend was observed for total cholesterol. No significant differences in HbA1c levels between deprivation quintiles were seen either before or after QOF [38].

The same cohort was used to calculate quality of care scores for diabetes patients, i.e. the number of achieved indicators divided by the number of indicators applicable to each patient [39]. The study found that diabetes care was improving already prior to QOF, and that the improvements accelerated during the first years of its implementation. Prior to the scheme, practices in deprived areas provided overall slightly better diabetes care compared to practices in affluent areas based on QOF indicators. However, patients in deprived areas gained less from QOF than patients from affluent areas during the early years of the scheme as practices in affluent areas seemed to have responded more quickly to the financial incentives introduced by QOF [39].

Using national surveys from year 2003 and 2006 in the UK, Crawley et al. compared the proportion of manual and non-manual workers with coronary heart disease (CHD), diabetes or hypertension that achieved target values of blood pressure, blood glucose and cholesterol [40]. Overall, the study found no difference in achievement of target levels for blood pressure or cholesterol levels between manual and non-manual workers in 2003 and 2006. Among diabetes patients, the usage of antihypertensive medicines, lipid-lowering drugs and oral hypoglycemic agents did not differ between the groups in either year. The proportion achieving target levels of HbA1c was significantly lower among manual workers than non-manual workers in 2003, but the gap was attenuated in 2006 [40].

### Cardiovascular disease

The proportion of patients that achieved target levels of indicators for cardiovascular disease improved significantly after the implementation of QOF in a serial cross-sectional study [41]. White patients had their blood pressure measured more frequently than south Asians and achieved target levels of blood pressure to a greater extent than blacks prior to QOF in 2003, but these differences were attenuated after the reform in 2005. In contrast, a higher proportion of south Asians achieved target levels of blood pressure in 2003, and the differences increased in 2005. Ethnic disparities in most cardiovascular indicators reduced after the introduction of QOF, although the between-year difference was not statistically significance [41].

Several studies assessed the impact of QOF on health care inequalities between socioeconomic groups [40, 42–44]. The above referenced study by Crawley et al. found that manual workers with CHD achieved blood pressure target levels to a lower extent than non-manual workers after the introduction of QOF, although no such difference existed prior to the scheme [40]. There were no significant differences in usage of antihypertensive medication or lipid-lowering drugs and in achieving target cholesterol levels between the two groups [40]. In a serial cross-sectional study of 310 general practices in Scotland, CHD patients from the least deprived quintile were more likely to receive influenza vaccination and to have smoking status and blood pressure recorded but less likely to receive anticoagulant therapy after the introduction of QOF compared to the most deprived quintile, while no such difference existed prior to QOF [42]. There were differences in prescription rates of ACE-inhibitors and beta-blockers between the deprivation quintiles prior to QOF, and the scheme did not change this difference [42]. Another study using Scottish data from 2001 to 2006 did not identify any difference between deprivation quintiles in the proportion of hypertensive patients achieving target values of blood pressure or in prescription practices of antihypertensive drugs, either before or after the introduction of QOF. Overall, the study observed improvements for all included parameters between 2001 and 2006, but did not account for the underlying trend [43].

Another serial cross-sectional study used four incentivized and ten non-incentivized quality indicators from QOF to compare hypertension management in individuals from areas with different deprivation scores between 2003 and 2005 [45]. Patients from more deprived areas achieved better results for one of the incentivised quality indicators (last blood pressure 150/90 or less) and two of the non-incentivised quality indicators (serum creatinine and electrolytes; electrocardiography) in 2003. Almost all indicators had improved by 2005; there were no differences by deprivation for the incentivised indicators, but two non-incentivised indicators (documented assessment of personal history of peripheral vascular disease; documented assessment of diabetes) differed significantly in favour of the more deprived population. Thus, patients from more deprived areas received at least equivalent quality of care as those from less deprived areas before the introduction of QOF, and QOF did not change this relation.

Simpson et al. studied patients with stroke or transient ischemic attack and found that registration of smoking status was more common in the most deprived than in the least deprived quintile, but the relation was reversed after the introduction of QOF [44]. Patients from the most deprived quintile were also less likely to receive anti-smoking advice or have their blood pressure recorded post-QOF. No differences between deprivation quintiles were observed for the use of MRI/CT scan, anticoagulant therapy, influenza vaccination, registration of cholesterol or proportion who achieved target cholesterol levels, either pre- or post-QOF [44].

An interrupted time series study by Lee et al. compared trends in mean blood pressure and mean total cholesterol level before and after QOF in patients with cardiovascular diseases [46]. QOF improved risk factor control for CHD, stroke and hypertension for all ethnic groups (black, south Asian and white), but did not contribute significantly to narrowing the gap between groups. In patients with coronary heart disease or stroke, the differences in systolic blood pressure between blacks and whites increased after the implementation of QOF.

### Respiratory diseases

In a serial cross-sectional study from the UK, the introduction of QOF and new clinical guidelines for COPD patients increased the registration of spirometry data and use of combination inhalers for all patients, with no differences between deprivation quintiles [47].

### Multiple diagnoses

Bhalla et al. studied the impact of pay-for-performance in a large, integrated health care delivery system in New York City. The providers were evaluated on a number of quality indicators that were grouped into five domains: diabetes, CHD, heart failure, screening and prevention, and all care [48]. Comparisons before and after the introduction of the pay-for-performance program were made within each racial group (American Indian/Alaskan Native, Asian, African American/black, Native Hawaiian/Pacific Islander, white and multiracial). The study found that the program improved diabetes care, screening and prevention care, and all care for all racial groups but Asians, who were receiving standard of care treatment to a higher extent already prior to the program. Analysis of interaction between race and year revealed no difference between groups in the effect of pay-for-performance. Caution should be applied in generalizing the results, as the health care system under study was more experienced in serving minority communities, who often received higher quality of care than their non-minority counterparts at start [48].

Dowd et al. compared mortality between HMO (capitation) and fee-for-service Medicare enrolees in the US, controlling for observed and unobserved confounders, and found no effect of health plan on mortality by either race/ethnicity (black vs. non-black, and Hispanic vs. non-Hispanic) or education [49].

### Preventive care

Based on cross-sectional data from Scotland in 2003–2004 and 2006–2007, influenza immunization uptake in the population aged ≥65 years and in clinical risk groups increased after the introduction of QOF, but pre-existing inequalities by deprivation status persisted [50].

A longitudinal cohort study of 4,284 patients in the UK found a significant increase in the proportion of diabetes patients whose smoking status was recorded and who received smoking cessation advice after the introduction of QOF [51]. With few exceptions, minority groups (black African, black Carribean, Indian, Pakistani, Bangladeshi) were significantly more likely to have their smoking status recorded and had larger increase in registrations than white British from pre- to post-QOF. There were no differences in the registration of smoking status between deprivation quintiles, or in the proportion receiving smoking cessation advice by ethnicity or deprivation [51].

## Discussion

This study did not find convincing evidence in favour of one reimbursement system over another with respect to impact on socioeconomic or racial inequalities in access, utilization and quality of primary care.

This review adds to existing literature by addressing the main types of reimbursement system and their impact on socioeconomic or ethnic/racial health care inequalities. The included studies covered a wide spectrum of outcomes, spanning over process measures to general and disease-specific outcomes. The heterogeneity of the outcomes complicates synthesis of results, since the reimbursement system might have differential impact on equity depending on the outcome under study. Patient characteristics other than race/ethnicity or socioeconomic status might also influence the effect of reimbursement systems and confound the results in these studies. For example, Taggar et al. found that the presence of chronic medical conditions was the strongest predictor associated with recording smoking status or cessation advice after the introduction of QOF [52]. Furthermore, the effect of a given reimbursement system might be context-specific and vary between different health care systems, such as universal tax-funded systems versus private health insurance systems. Our review was dominated by studies from the US and the UK, which limits generalizability and comparability between studies since the structure of the health systems differ widely between these countries. Additionally, studies from the US may be more vulnerable to selection bias since patients choose their insurance plan. The dominance of studies from the US and the UK might be a result of the English language restriction in this review, and studies from other countries addressing policy-related research questions might be more commonly published in the form of gray literature.

It is debatable whether the outcome measures in the included studies are sensitive to socioeconomic and racial inequalities. For example, changes in laboratory values or blood pressure might be insufficient in capturing inequalities in health care utilization and quality of care. A reason for choosing these metrics could be data accessibility; many of them are included in the QOF scheme and tracked over time. However, this implies that other important outcomes that could be suitable for measuring inequity were neglected. For example, none of the studies in this review addressed mental health that disproportionately affects the lower socioeconomic groups. Furthermore, none of the studies distinguished between horizontal and vertical equity [53]. Observing equal numbers of physician visits among different groups might not imply equal health care utilization if the need for health care differs between groups, and similarly unequal numbers might not imply inequity. In most studies, a combination of different reimbursement systems (capitation, fee-for-service and pay for performance) was applied, making it difficult to disentangle the specific effect of one type of reimbursement system over another. Additionally, adjustment of reimbursement by e.g. age and socioeconomic factors in the population to account for differential health care need between areas was not accounted for and might have occurred in some studies but not others, further contributing to the complexity of reimbursement systems. Changes in reimbursement systems are often accompanied by the implementation of other measures, such as new guidelines, additional investments in healthcare and organizational changes, which complicates causal inference. Thus, any improvements observed may or may not be attributed to the reimbursement system per se. A caveat of studying health care reforms are the natural changes that would have occurred over time regardless of the reform, which could be addressed by taking time trends into account, but few studies applied this method.

In spite of the lack of conclusive findings, this review illustrates the complexity of reimbursement systems, and the need of disentangling separate stages at which inequalities in health care may appear. The study also illustrates the lack of scientific evidence supporting the impact of reimbursement systems on equity in primary care, which in one sense is surprising given the many reforms that involve changes in reimbursement systems. The recent primary care reform in Sweden, with variations of the reimbursement system implemented in twenty county councils and regions, would provide a unique opportunity to study the effects of reimbursement on equity in access and quality of care within the same context and national health system. Ultimately, it may be the effect of reimbursement system on resource allocation that has the greatest impact on health care inequity. Barr et al. demonstrated how need-based resource allocation to deprived areas in the UK reduced health inequities [54]. The rate of mortality amenable to health care interventions declined faster in areas receiving the need-based resource allocation than in other areas. This difference in the rate of decline was not observed for all-cause mortality, suggesting that additional resources to deprived areas were translated into health care services that benefitted those with greater needs. Hence, resource allocation that matches the increased health care needs of underserved might have a greater impact on health inequalities than the type of reimbursement system per se.

## Conclusions

The choice of reimbursement system seems to have limited impact on socioeconomic or racial inequity in access, utilization and quality of primary care. The lack of conclusive evidence may be partly explained by the complexity of reimbursement systems, the limitations in study design and the context-specific findings. To reduce health care inequalities, policy makers may consider other strategies in addition to reforming the reimbursement system, such as need-based resource allocation to underserved populations. Further empirical studies are warranted that address how resource allocation and reimbursement systems should be designed in order to best serve those with greater health care needs and reduce health care inequalities.

## Additional files

Additional file 1: Search strategy in Web of Science and PubMed, including date and search results. (DOCX 12 kb)
Additional file 2: Data extraction form for studies included in the systematic review. (DOCX 12 kb)
Additional file 3: Summary of the 22 studies in included in the systematic review. (DOCX 19 kb)

## Abbreviations

ACE-inhibitors: Angiotensin-converting enzyme inhibitors
ACSC: Ambulatory care sensitive conditions
AHA: Antihypertensive agents
BP: Blood pressure
CHD: Coronary heart disease
FFS: Fee-for-service
HMO: Health maintenance organization
OHA: Oral hypoglycaemic agents
P4P: Pay-for-performance
PCP: Primary care practitioner
QOF: Quality and outcomes framework
SEP: Socioeconomic position
UK: United Kingdom
US: United States
