# Supplementary material for: The impact of reimbursement systems on equity in access and quality of primary care: A systematic literature review
Source: BMC Health Serv Res. 2016 Oct 4;16:542. doi: 10.1186/s12913-016-1805-8 (PMC5050924; doi:10.1186/s12913-016-1805-8)
Supplement: Additional file 1: — Search strategy in Web of Science and PubMed, including date and search results. (DOCX 12 kb) [file 12913_2016_1805_MOESM1_ESM.docx]

**Additional file 1. Search Strategy**

**Web of Science**

Topic=(reimburs* OR remunerat* OR "pay for performance" OR "fee for service" OR capitat* OR salar* OR Quality and Outcomes Framework OR payment method* OR payment system* OR incentive*)

AND

Topic=(inequalit* OR inequit* OR equalit* OR equit* OR disparit* OR socioecon* OR social class* OR ethnic* OR race OR racial OR accessibilit* OR utiliz*)

AND

Topic=( "family practice*" OR "general practice*" OR "primary health care" OR "primary care")

**PubMed**

("Reimbursement Mechanisms"[Mesh] OR "Physician Incentive Plans"[Mesh] OR "Fee for service"[All Fields] OR "Pay for performance"[All Fields] OR "Capitation"[All Fields] OR "Incentives"[All Fields] OR "Remunerate"[All Fields] OR "Remuneration"[All Fields] OR "Payment method"[All Fields] OR "Payment system"[All Fields] OR "Payment methods"[All Fields] OR "Payment systems"[All Fields])

AND

("Quality of Health Care"[Mesh] OR "Quality Indicators, Health Care"[Mesh] OR "Outcome Assessment (Health Care)"[Mesh] OR "Health Status Disparities"[Mesh] OR "Socioeconomic Factors"[Mesh] OR "Continental Population Groups"[Mesh] OR "Ethnic Groups"[Mesh] OR "ethnicity"[All Fields] OR "ethnic"[All Fields] OR "race"[All Fields] OR "Inequalities"[All Fields] OR "Inequality"[All Fields] OR "Inequities"[All Fields] OR "Inequity"[All Fields] OR "Equity"[All Fields] OR "Equality"[All Fields] OR "disparities"[All Fields] OR "social class"[MeSH Terms] OR "social class"[All Fields] OR accessibility[All Fields] OR "utilization"[All Fields])

AND

("Primary care physician"[All Fields] OR "Primary care"[All Fields] OR "Primary health care"[All Fields] OR "General Practice"[Mesh] OR "Primary Health Care"[Mesh] OR "Physicians, Primary Care"[Mesh])

Results from search in Web of Science without filter on September 30, 2013: 790 abstracts

Results from search in Web of Science with language filter (English) on September 30, 2013: 765 abstracts

Results from search in PubMed without filter on September 30, 2013: 3087 abstracts

Results from search in PubMed with language filter (English, Swedish) and year filter (1980-2013) on September 30, 2013: 2890 abstracts
