# Supplementary material for: The impact of reimbursement systems on equity in access and quality of primary care: A systematic literature review
Source: BMC Health Serv Res. 2016 Oct 4;16:542. doi: 10.1186/s12913-016-1805-8 (PMC5050924; doi:10.1186/s12913-016-1805-8)
Supplement: Additional file 2: — Data extraction form for studies included in the systematic review. (DOCX 12 kb) [file 12913_2016_1805_MOESM2_ESM.docx]

**Additional file 2: Data Extraction Form for the Studies included in the Systematic Review**

| **Heading** | **Description** |
| --- | --- |
| Author, year | Authors and year of publication |
| Journal | Title of journal |
| Study Design | Randomized trials, cohort study, case-control study, ecological study etc. |
| Population | Size of study sample |
| Time period | Time period under study |
| Cohort/database | Data sources and, if applicable, name of cohort from which study population is derived |
| Inclusion/exclusion criteria | Inclusion and exclusion criteria into the study |
| Country/state | Country/state from where the study participants were recruited |
| Reimbursement system | Fee-for-service, capitation or pay-for-performance |
| Equity aspect | Race/ethnicity or socioeconomic |
| Primary outcome | Primary outcome of study |
| Secondary outcome(s) | Secondary outcome(s) of study |
| Statistical analysis | Statistical methods used and effect measurement |
| Results | Summary of main results relevant for the systematic review |
| Conclusion | Overall conclusion from the study |
