# Supplementary material for: The impact of reimbursement systems on equity in access and quality of primary care: A systematic literature review
Source: BMC Health Serv Res. 2016 Oct 4;16:542. doi: 10.1186/s12913-016-1805-8 (PMC5050924; doi:10.1186/s12913-016-1805-8)
Supplement: Additional file 3: — Summary of the 22 studies in included in the systematic review. (DOCX 19 kb) [file 12913_2016_1805_MOESM3_ESM.docx]

**Additional file 3.** Summary of 22 studies included in the systematic review

| **Author, year** | **Setting, population** | **Intervention** | **Equity** | **Outcome** | **Key findings** | **Study quality** |
| --- | --- | --- | --- | --- | --- | --- |
| Stepanikova 2004 [30] | USA, 1998-2000 n=7,213 adults from 48 states. Cross-sectional. Patient level survey data. | Capitation and non-capitation | Ethnicity/race | Patient satisfaction during primary care visit: how well the physician listened and explained; how thorough the physician was during examination | Black, Hispanic, and Native American/Asian/Pacific Islander reported lower patient satisfaction than whites in a capitated systems, but the differences were only statistically significant for English-speaking Hispanics. **Capitation 0/-** | Medium |
| Le Cook 2007 [10] | USA, 1997-2001 n=3,286 Medicaid enrolees. Cross-sectional. Survey and administrative data. | Capitation and FFS | Ethnicity/race | Patient-reported access and utilization of care: having a usual source of care, doctors visit and ER use during last year | Disparities between whites and blacks or Hispanics in access and utilization of care were lower among Medicaid managed care beneficiaries than Medicaid FFS enrolees. The difference was were especially pronounced for blacks. **Capitation +** | Medium |
| Balsa  2007 [31] | USA, 1996-2001  n=52,781 Medicare enrolees. Cross-sectional. Survey data. | Capitation and FFS | Ethnicity/race | Patient-reported access and utilization of care: having a usual source of care, doctors visit, delay of care due to cost, receiving essential medical care | Compared to whites, managed care reduced inequalities in having a usual source of care for Hispanics, seeing a health professional for blacks, and out-of-pocket costs for blacks and Hispanics. **Capitation +** | Medium |
| Muggah  2012 [32] | Canada, 2005-2006. n=5,269 adults in Ontario. Cross-sectional. Survey data. | Capitation, FFS and salaried | Ethnicity/race | Patient-reported access and utilization of primary care: no. of visits to primary care, first contact access, first contact utilization | Immigrants consumed more primary health care than Canadian-born. However, recently arrived immigrants (<5 years) in FFS practices did not consume more healthcare and experienced inferior access to primary care compared to Canadian-born. The differences were less pronounced for capitated practices. **Capitation +** | Medium |
| Bindman  2005 [9] | USA, 1994-1999 n=2.4 million adult Medicaid enrolees in California. Cross-sectional. Administrative data | Capitation and FFS | Ethnicity/race | Admissions for ambulatory care sensitive conditions (ACSC) | Managed care patients had lower rates of ACSC admission than FFS patients, and the differences were more pronounced in African American, Asian and Latino groups than in whites. Both the rate of new admission and readmission were decreased in managed care patients, indicating improved access to care as well as quality of care. **Capitation +** | High |
| Alshamsan  2012 [35] | UK, 2000-2007 n= 7,434 diabetes patients in Southwest London. Longitudinal cohort. Medical records. | P4P | Ethnicity/race | Mean BP, total cholesterol, HbA1c | Existing ethnic disparities between whites, blacks and South Asians remained largely intact after the implementation of P4P, and P4P did not seem to add any benefit to the pre-existing downwards going trend for mean HbA1c, total cholesterol and systolic blood pressure observed in patients of all ethnicities. **P4P 0** | Medium |
| Millett  2007 [36] | UK, 2003, 2005 n= 4,284 diabetes patients in Southwest London. Longitudinal cohort. Medical records | P4P | Ethnicity/race | Proportion achieving target values of HbA1c, BP, total cholesterol. Prescriptions (insulin, OHA, ACE-inhibitor, lipid-lowering agents) | Target values of HbA1c, BP and total cholesterol, and prescriptions of medications improved across all ethnicities (black African, Indian, Pakistani, Bangladeshi, white British, white Irish) after the implementation of P4P, except for black Carribeans who lagged behind in HbA1c and BP control. **P4P 0** | Medium |
| McGovern  2008 [37] | UK, 2004, 2005 n=37,329 and 56,561 diabetes patients in Scotland in 2004 and 2005, respectively. Cross-sectional. Administrative data. | P4P | SEP | Registration and proportion achieving target levels of HbA1c, total cholesterol, BP, and creatinine. | Diabetes patients from the most deprived quintile are less likely to achieve target HbA1c levels, and P4P did not reduce this inequality. The most deprived quintile were more likely than the least deprived quintile to achieve target cholesterol levels after the implementation of P4P, whereas the reversed was observed for registration of BP levels. **P4P 0** | Medium |
| Hamilton  2010 [38] | UK, 1997-2005 n= 154,945 diabetes patients. Longitudinal cohort. Medical records. | P4P | SEP | Registration and achievement of target levels of HbA1c, total cholesterol, BP. Prescriptions (OHA, AHA, lipid-lowering agents) | Proportion achieving target values of HbA1c, BP and total cholesterol after the introduction of P4P improved for all, but did not impact on inequalities between deprivation quintiles. Prescription of medication differed significantly between deprivation quintiles both in 1997 and 2005. **P4P 0** | High |
| Kontopantelis  2013 [39] | UK, 2000-2007 n=23,780 diabetes patients. Longitudinal cohort. Medical records. | P4P | SEP | Composite of quality of care score - percentage of indicators achieved compared to the total number of indicators (17 diabetes indicators) | Patients from the most deprived quartile of practices gained less benefits from P4P than patients from the most affluent quartile of practices. **P4P -** | High |
| Crawley  2009 [40] | UK, 2003, 2006 Patients with diabetes (n=661 and 562), CHD (n=861 and 557) and hypertension (n=3,717 and 2,996) in 2003 and 2006, respectively. Cross-sectional. Survey data. | P4P | SEP | Registration of target values of HbA1c, BP, total cholesterol. Prescriptions (OHA, AHA, lipid-lowering agents) | Manual workers with CHD achieved target BP values to a lower degree than non-manual workers under P4P, although no such difference existed prior to the scheme. In contrast, inequalities in target HbA1c levels between manual and non-manual workers with diabetes attenuated after the implementation of P4P. No significant difference was found for the other parameters. **P4P 0** | Medium |
| Millett  2009 [41] | UK, 2003, 2005 n=2,891 and 3,101 CHD patients in 2003 and 2005 respectively. Cross-sectional. Administrative data. | P4P | Ethnicity/race | Registration of BMI, smoking status. Prescription of AHA, lipid-lowering agents, aspirin. Registration and achievement of target values of BP and cholesterol. | South Asians seems to have benefitted most from P4P, and were more successful in achieving some of the indicators than whites. Difference in BP control between blacks and whites were attenuated after the introduction of P4P, whereas inequalities in statin prescription remained unchanged. **P4P +** | Medium |
| McGovern  2008 [42] | UK, 2004, 2005 n=58,406 and 75,495 patients with CHD in 2004 and 2005, respectively. Cross-sectional. Administrative data. | P4P | SEP | Registration and achievement of target values of CHD-related quality indicators: smoking, exercise test, BP, cholesterol, prescription, influenza vaccination. | Influenza vaccinations, registration of smoking and BP status, and prescription of beta-blockers were more common in the least deprived quintile than in the most deprived quintile after the implementation of P4P. In contrast, anticoagulant therapy and ACE-inhibitors were more frequently prescribed in the most deprived quintile. **P4P 0/-** | Medium |
| Simpson  2011 [43] | UK, 2001-2006 n=142,976 and 199,207 hypertensive patients in 2001 and 2006, respectively. Cross-sectional. Administrative data. | P4P | SEP | Registration and achievement of target value of BP. Prescription of AHA. | After the implementation of P4P, most deprived patients were less likely to have their BP recorded than the least deprived patients. There were no other significant differences between deprivation quintiles before and after P4P. **P4P -/0** | Medium |
| Simpson  2006 [44] | UK, 2004, 2005 n=21,901 and 32,401 patients in 2004 and 2005, respectively. Cross-sectional. Administrative data. | P4P | SEP | Registration and achievement of target values of stroke-related quality indicators: diagnosis, smoking, BP, cholesterol, prescription, influenza vaccination. | Smoking status was more frequently registered in the most deprived quintiles compared to the least deprived quintile before the implementation of P4P, but the relationship became reversed with the new scheme. P4P also resulted in fewer registrations of BP among the most deprived quintile, while no such differences existed prior to P4P. **P4P -** | Medium |
| Hammouche  2011 [45] | UK, 2003, 2005  n=304 patients with hypertension and at least one record of systolic BP >150 or diastolic BP>90. Cross-sectional. Medical records. | P4P | SEP | Four incentivised quality indicators from QOF and ten non-incentivised quality indicators | For most indicators there has been a positive effect of QOF on achievements. Overall, QOF did not affect disparities. Population in more deprived areas received at least the same level of quality of care as populations in less deprived areas both before and after QOF. **P4P 0** | Medium |
| Lee  2011 [46] | UK, 2000-2007  n=1 753, 2 952 and 15 035 patients with stroke, CHD and hypertension respectively.  Longitudinal cohort.  Medical records. | P4P | Ethnicity/race | Target value of BP and total cholesterol | P4P improved risk factor control for CHD, stroke and hypertension in all ethnic groups (black, south Asian and white), but differences in BP between blacks and whites with CHD or stroke increased after the implementation of P4P. **P4P -** | High |
| Smith  2008 [47] | UK, 2003, 2005 n=25,565 and 29,870 COPD patients in 2003 and 2005, respectively. Cross-sectional. Administrative data. | P4P | SEP | Registration of spirometry. Prescription of combination inhalers. | There were no differences between deprivation quintiles in the proportion of patients with recorded spirometry data and prescription of combination inhalers before or after the implementation of P4P. **P4P 0** | Medium |
| Bhalla  2013 [48] | USA, 2007, 2009 n=5,824 randomly selected patients, Bronx. Cross-sectional. Medical records. | P4P | Ethnicity/race | 26 quality indicators in five domains (diabetes, CHD, heart failure, screening/prevention, all-care). Proportion of recommended care received. | There were improvements in all care, diabetes care, and screening/preventive care after the implementation of P4P. All ethnicities (American Indian/Alaskan Native, African American/black, Native Hawaiian/Pacific Islander, white and multiracial) benefitted from the new scheme, except for Asians who received standard of care to a higher extent than other groups prior to P4P. **P4P 0/+** | Medium |
| Dowd  2011 [49] | USA, 1996, 1998  n=15 164 Medicare enrollees. Longitudinal cohort.  Survey and administrative data. | Capitation and FFS | SEP and ethnicity/  race | 2-year predicted mortality | Patients under a capitated reimbursement plan had lower predicted mortality than FFS patients. Race (black vs. non-black), ethnicity (Hispanic vs. non-Hispanic), poverty and education did not significantly contribute to the predicted mortality. **Capitation 0** | Medium |
| Norbury  2011 [50] | UK, 2003, 2006 n=327,423 and 358,330 in 2003 and 2006, respectively. Cross-sectional. Administrative data. | P4P | SEP | Influenza vaccination uptake among eligible patients. | Influenza vaccination uptake among eligible patients decreased with increasing deprivation in area of residency, and P4P did not impact on this inequality. **P4P 0** | Medium |
| Millett  2007 [51] | UK, 2003, 2005 n=4,284 diabetes patients. Longitudinal cohort. Medical records. | P4P | SEP and ethnicity/ race | Registration of smoking status, smoking cessation advice, smoking prevalence. | Smoking status are more frequently registered in black African, black Carribean, Indian, Pakistani and Bangladeshi than in white British. There was no difference between ethnicities in the proportion receiving smoking cessation advice. There were no differences between deprivation quintiles either before or after P4P. **P4P 0/+** | Medium |

FFS= Fee-for-service; PCP=Primary care practitioner; P4P=Pay for performance; SEP=Socioeconomic position; BP=Blood pressure; OHA=Oral hypoglycaemic agents; ACE=Angiotensin-converting enzyme; AHA=Antihypertensive agents; CHD=Coronary heart disease. “0” indicates no difference in inequity, “-“ indicates greater inequity, “+” indicates lesser inequity, “0/+” and “0/-“ indicate that results were mixed depending on outcome and/or socioeconomic or ethnic/racial group.
